# Exploring the Intersection of Rare Diseases and Mental Health Within the Diagnostic Odyssey: A Narrative Review and Thematic Synthesis

**DOI:** 10.1002/nop2.70488

**Published:** 2026-04-02

**Authors:** Eileen Wu, Sophie Isobel, Paul Beckett

**Affiliations:** ^1^ Susan Wakil School of Nursing and Midwifery, University of Sydney Sydney New South Wales Australia

**Keywords:** health services, lived experience, mental health, psychological, rare diseases, resilience

## Abstract

**Aim:**

To explore what is known about the intersection of mental health and rare diseases.

**Design:**

Narrative review with peer‐reviewed literature from 2009 onwards.

**Methods:**

The study searched for literature on these databases in September 2024: CINAHL, Scopus, Pubmed, Medline, Embase, and PsycInfo, as well as citation chaining and supplementary searches on Google Scholar. A combination of MeSH headings, keywords, truncations, and proximity searches with Boolean operators was used.

**Results:**

Relevant literature highlighted four themes that underpinned the intersection of mental health and rare diseases: (1) hope and hopelessness, (2) identity formation and conflicts, (3) connection and disconnection, and (4) access, advocacy, and a lack of service integration.

**Conclusion:**

The narrative review highlighted the complex intersection and poor integration of mental health and rare diseases, where distress and uncertainty are core aspects of the living experience. Despite challenges, hope and resilience persevere.

**Implications:**

Better understanding of the connection between mental health and rare diseases allows for holistic patient care. It raises awareness for the need for increased proactive mental health services and highlights shared experiences of a broad community. This allows systemic changes to be more feasible and help improve patient outcomes.

**Impact:**

This narrative review deepens knowledge of the complex connections between mental health and rare diseases through a lived experience lens, opening pathways for further research into the unique but shared struggles of the community. Emphasising the need for holistic disease management of physical and psychosocial impacts, nurses and healthcare workers alike are then better equipped to provide tailored care.

**Patient or Public Contribution:**

There was no patient or public contribution to this paper as the review utilised existing studies and research in the academic field.

## 1 Introduction

Rare diseases (RD) encompass a broad range of conditions that affect fewer than one in 20,000 people (Rare Disease International, n.d.). They are often genetic, long‐term, multisystemic, and significantly detrimental to quality of life (Rare Disease International, n.d.). Despite the rarity of these conditions, the cumulative impact of up to 10,000 known diseases affects approximately 300 million people worldwide (Haendel et al. 2020; Rare Disease International, n.d.). Although there is heterogeneity across the spectrum of RD, there are also common experiences. RD often have no effective cure, leading to lifelong complex treatment to relieve symptoms (Llubes‐Arrià et al. 2022). Hence, the experience for people with RD is often affected by physical, financial, functional and emotional burdens which can lead to mental distress (Molster et al. 2016; Bauskis et al. 2022).

Mental health is the “state of mental well‐being that enables people to cope with the stresses of life, realise their abilities, learn and work well, and contribute to their community.*”* (World Health Organisation 2022). Poor mental health and psychological stress are associated with reduced quality of life, lower life satisfaction, and diminished well‐being (Bryson and Bogart 2020). Common experiences in people with RD, such as lack of psychological support, disease burden, social discrimination and high stress, contribute to decreased mental well‐being (Bryson and Bogart 2020). A unique experience that impacts mental health is their diagnostic odyssey—a term coined in academic literature to describe the long and complex process required to receive a correct diagnosis. The odyssey often involves delays, misdiagnoses, and invasive investigations, contributing to financial, physical and psychosocial burdens (Byun et al. 2022; Bauskis et al. 2022). As a consequence, individuals are at increased risk of developing symptoms of anxiety and depression, further contributing to reduced mental health and quality of life (Sánchez‐García et al. 2021).

The challenges faced by people with RD have been explored in individual studies, but a synthesis of lived accounts is lacking (Llubes‐Arrià et al. 2022; Bauskis et al. 2022; Kenny and Stone 2022). Furthermore, while some studies have examined the experience of living with an RD, there is a lack of focus on mental health. As such, little is known about the intersection of RD and mental well‐being, particularly with regard to mental health service access and needs. Existing research that explores the psychosocial impact and burden on individuals also focuses heavily on quantitative methodology (von der Lippe et al. 2017). Moreover, a biomedical deficit‐based approach persists in existing literature despite recognition of the psychosocial impact and burden of RD on well‐being. As a result, there is less understanding of the resilience, strengths, and coping of individuals with RD and mental health recovery, as well as a lack of clarity about how to provide effective psychosocial support (Byun et al. 2022). A comprehensive overview of the intersection of mental health and RD through lived experiences is absent.

## 2 Aim

This narrative review aims to address the gap in literature by exploring and synthesising what is known about the intersection of mental health and RD. The aim was iteratively refined throughout the review with the following guiding questions for the search:

1. What is known about people with RD's experience with mental distress?
2. What is known about the impact of RD on mental health throughout the diagnostic odyssey?
3. What is the function of mental health support for people with RD?

## 3 Method

### 3.1 Study Design

A narrative review using systematic searching was undertaken. Narrative reviews allow interpretation and critical literature synthesis to construct a meaningful ‘narrative’ of evidence surrounding complex experiences (Greenhalgh et al. 2018). While narrative reviews vary in their approaches, they can be undertaken using systematic methods and a clear criterion to enhance validity, while enabling a thorough and nuanced exploration of the literature (Green et al. 2006). A narrative review was appropriate for this study as it allowed for an in‐depth exploration of the intersection of mental health and RD, centred around people's experiences. Much of the relevant literature is qualitative or opinion articles that may not meet the criteria for systematic reviews. Hence, the narrative method allowed for interpretation, reflection, and critical synthesis of literature relevant to this under‐researched area—while accommodating subjectivity and diverse perspectives (Sukhera 2022).

### 3.2 Search Methods

A systematic search strategy was designed in consultation with a specialist librarian, with search terms and criteria refined over time. Searches were undertaken in Cumulative Index to Nursing and Allied Health Literature (CINAHL), Scopus, Pubmed, Medline, Embase, and PsycInfo. MeSH headings, keywords, truncations, and proximity searches were used with Boolean operators “and” and “or”. Search terms included “rare disease”, “mental health”, “lived experience”, “perspective” and other similar terms (see Appendix A).

Citation chaining also occurred, as well as supplementary searches through Google Scholar.

### 3.3 Eligibility Criteria

Inclusion and exclusion criteria were constructed in line with the study aim (see Table 1). One author screened eligible articles by title, abstract, and then full text. Other authors were involved in the consultation process of article selection based on the inclusion criteria and research questions. This occurred simultaneously with the writing process. As the review aimed to use lived experiences to inform the understanding of the intersection between mental health and RD, studies that involved quantitative measures only were excluded. Since the experience of caregiver burden on mental health is well documented, articles that did not include RD individuals as the main participants and instead focused on caregivers or families only were excluded (Buckle et al. 2024). This allowed studies to be more centralised on the lived experiences of RDs and their impact on mental health. Papers not published in English were also excluded.

**TABLE 1:** Inclusion and exclusion criteria.

Include	Exclude
About RD Focused on lived experience relating to mental health Involved people with RD as main participants or authors	Not in English Studies with quantitative measures only Reviews, book chapters, conference abstracts Not peer‐reviewed Full text unavailable

**FIGURE 1:**
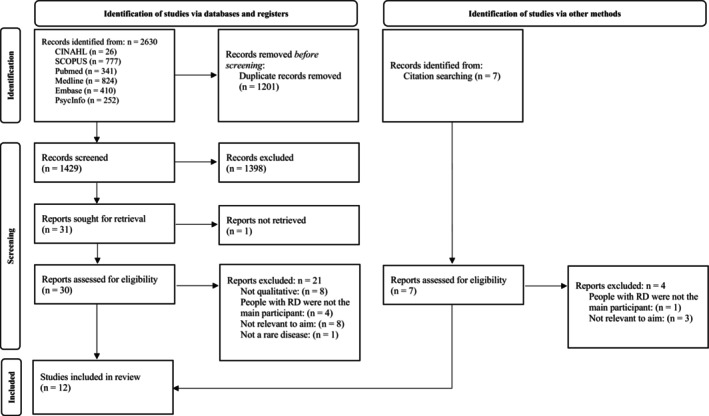
PRISMA Flow Diagram.

Of the 2630 articles that arose from database searching, 1429 remained after removing duplicates. After screening titles, abstracts, applying the inclusion/exclusion criteria and reviewing relevance to the study, 12 studies were included in the review (see Figure 1).

### 3.4 Quality Appraisal

Narrative reviews aim to provide an interpretative overview of the topic by drawing from diverse experiences and sources, rather than relying on the highest quality of evidence (Sarkar and Bhatia 2021). In line with the review search strategy, all articles included were peer‐reviewed and published in academic journals. While all articles had clear aims and objectives, the sparsity of knowledge on the topic meant that lived experiences were drawn from qualitative studies, editorial pieces written by people with RD, and case studies. While the small sample sizes of included studies may allude to inaccuracy and bias, qualitative methodologies focus on gathering a range of perspectives and experiences rather than ensuring applicability to the whole population (Braun and Clarke 2021). The limited number of available studies on the topic meant that articles were from across the globe, published between 2009 and 2024. The small number of relevant studies identified through the search and the heterogeneous nature of the included articles indicate a need for further research to build a robust evidence base that reflects the experiences of people living with these conditions. Whilst established hierarchies of evidence prioritise systematic reviews, exploring the lived experiences of people with health conditions remains crucial for progressing effective and meaningful research and practice (Beames et al. 2021).

### 3.5 Synthesis

Analysis of included articles occurred iteratively, with consistent reflection on the study aims and emergent themes. A thematic synthesis was undertaken to identify patterns and link ideas to form a narrative. Following Thomas and Harden's three‐stage approach (2008), the study findings were repeatedly read to identify key findings, which were then organised into related areas to construct descriptive themes for analysis. The themes were discussed amongst the authors until overarching interpretive themes were agreed upon. The themes were developed to address the aim of understanding how the experiences described in the literature inform the intersection between RD and mental health.

## 4 Results

12 articles were included in the review (see Table 2). The review totals 570 lived experiences, and excluding a qualitative analysis of a survey of 384 responses, the number of participants varied from 1 to 89. Studies were from across the globe, including the United Kingdom, Germany, France, Ireland, United States of America, Turkey, Japan, and Australia. Identified formats of studies that explored lived experiences included letters to the editor, case reports, qualitative, and mixed methods studies.

**TABLE 2:** Overview of studies included in review (*n* = 12).

Author	Country	Study type	RD	Aim	Results
Nunn (2017)	UK	Letter to the editor	N/A	To explore how a rare disease impacts on an individual's life and how these impacts are related to factors that also predispose to mental health difficulties	N/A
Witt et al. (2023)	Germany	Qualitative study—semi‐structured interviews (*n* = 89, including parents and children)	Collection of different RDs (unspecified)	To identify the needs, obstacles, barriers in families with children with a rare disease	Five main themes: Daily life with an RD The experiences with the healthcare system Psychosocial support Difficulties and barriers Improvements for patient‐oriented support
Zhang (2023)	UK	Letter to the editor	Ehlers‐Danlos syndrome	To compare and contrast different diagnostic journeys of rare diseases, and to reflect on its impact on the mental wellbeing of a patient	N/A
Uhlenbusch et al. (2019)	Germany	Qualitative study—focus groups (*n* = 18 adults)	Neurofibromatosis type 1 Primary sclerosing cholangitis Pulmonary arterial hypertension Marfan syndrome.	To explore the perceived burden of patients with rare diseases, and identify common and different experiences amongst patient with different conditions	Five main themes: Medical problems Psychological burden Problems concerning healthcare system Constraints Interpersonal problem
Buckle et al. (2024)	Ireland	Qualitative study – semi‐structured interviews (*n* = 11 children or young people)	Phenylketonuria Gaucher disease Dystonia Homocystinuria Hemolytic uremic syndrome Cystinosis	To gain insight into the daily challenges of children living with rare diseases.	Two themes with corresponding subthemes: Knowledge and understanding of rare diseases Fitting in versus Feeling different
Henderson et al. (2009)	US	Qualitative study—semi‐structured interviews—and quantitative measures (*n* = 17, including adults, adolescents and parents)	Niemann‐Pick Disease Type B	To gain deeper understanding of the rich experience of living with a chronic illness, and to assess the psychosocial adjustment of these patients	Five main themes: Limited physical activity, social isolation, peer rejection Close family relationships as a way of coping Impact of RD on psychosocial development Frustration regarding lack of information and treatment Psychosocial impact of medical experiences
Brugallé et al. (2021)	France	Qualitative study—semi‐structured interviews (*n* = 10 adults)	Holt‐Oram syndrome	To highlight people's subjective perceptions of their condition and its impact on providing meaning. To highlight the consequences of rare diseases on daily life and throughout life stages	Two main themes with corresponding subthemes: Stages of self‐construction as different When I am no longer the only one involved
Takeuchi et al. (2016)	Japan	Qualitative study—semi‐structured interviews (*n* = 6 adults)	Idiopathic basal ganglia calcification 3 (IBGC3)	To describe the lives and illnesses of people with IBGC3	Six themes: Frustration and anxiety with progression of symptoms without being diagnosed Confusion about diagnosis with an unfamiliar disease Emotional distress caused by a genetic disease Passive attitude towards life, being extra careful Taking charge of life, becoming active and engaged Requests for healthcare
Oksel (2020)	Turkey	Case report	Systemic sclerosis	To explore the psychosocial aspect and unseen impacts of scleroderma	Two guiding themes: Living with the disease Thoughts about the future
Hanson et al. (2018)	Australia	Qualitative study—semi‐structured interviews (*n* = 20 children or adolescents)	Primary lymphoedema	To describe the experiences and views of patients with lymphoedema	Six main themes with corresponding subthemes: Reinforcing abnormality Vulnerability and caution Negotiating uncertainties Developing resilience Taking responsibility Disruptive transition
Bogart et al. (2012)	US	Qualitative study—focus group (*n* = 12 adults)	Moebius Syndrome	To explore the experiences and strategies for social interaction from the perspective of individuals with Moebius Syndrome	Five themes: Social engagement/disengagement Resilience/sensitivity Social support/stigma Being understood/misunderstood Public awareness/lack of awareness of Moebius Syndrome
Munro et al. (2022)	US	Qualitative analysis of survey (*n* = 384 adults with one or more RDs)	Collection of different RDs (unspecified)	To focus on the stigma in individuals across different types of rare diseases.	Three main themes with corresponding subthemes: Structurally enacted stigma Interpersonally enacted stigma Felt stigma

While some articles focus on a specific RD, this review will refer to all RD participants collectively, regardless of diagnosis. Four overarching themes were identified: (i) hope and hopelessness; (ii) identity formation and conflict; (iii) connection and disconnection; and (iv) access, advocacy and a lack of integration. Results are presented under each interpretive theme.

### 4.1 Hope and Hopelessness

The intersection of mental health and RD includes a balance between experiences of hope and hopelessness. For people living with RD, life is often stressful and unpredictable, with concerns and uncertainty related to disease progression, lack of diagnosis, career impacts and family planning (Nunn 2017). Anxiety, loss, and hopelessness are consistent across these experiences, leading to mental health challenges. Yet, these experiences also contribute to the development of resilience and tenacity, as individuals develop ways to maintain hope despite challenges (Nunn 2017; Witt et al. 2023; Uhlenbusch et al. 2019; Takeuchi et al. 2016; Hanson et al. 2018).

While RD can impact mental health, people with RD may also have diagnosed mental disorders that are pre‐existing or exacerbated by their condition (Zhang 2023). Uhlenbusch et al. (2019) found that individuals often experience low mood, rumination, avolition, suicidality, pessimism, and self‐doubt. These experiences are compounded by physical and mental RD symptoms and can lead to a variety of mental health diagnoses like anxiety or depression (Uhlenbusch et al. 2019; Zhang 2023). RD that result in visible bodily changes or abnormalities also exacerbate shame, low self‐esteem, negative body image, despair, and hopelessness (Uhlenbusch et al. 2019; Munro et al. 2022; Oksel 2020). Complex grief can occur due to lost opportunities to live a normal life, as well as the trauma of losing loved ones due to genetic RD (Oksel 2020; Zhang 2023).

People with RD often experience hopelessness and uncertainty about the future. Anxiety about care needs and the possibilities of deteriorating health and functioning can lead to a pessimistic outlook on life (Nunn 2017; Uhlenbusch et al. 2019). To distance self from the chronicity of RD, some individuals adopt a ‘passive attitude’ towards life by being more conscious of not causing ‘trouble’ for others and hiding symptoms, compounding isolation and compromising their well‐being (Takeuchi et al. 2016). In the face of uncertainty, out of fear of being perceived as weak or inadequate, individuals may adopt restrictive behaviours, excessive conscientiousness, or may not engage in preventative health‐related activities as attempts to fit in or regain control (Henderson et al. 2009; Hanson et al. 2018).

Despite the challenges of living with an RD, many individuals remain hopeful by refusing to be limited by their disease, accepting their condition, and actively managing their lives and health (Takeuchi et al. 2016; Buckle et al. 2024). When health professionals provide limited information or support, people often take the initiative to seek out health information themselves, fostering empowerment and proactive self‐management (Takeuchi et al. 2016). Diagnostic confusion often requires those with RD to become their own advocates and, instead, educate the health professionals (Munro et al. 2022). Efforts to shift the focus away from their RD see individuals working on different areas of their lives that are within their control, as they strive to be high‐achieving, empathetic, tolerant, and determined (Brugallé et al. 2021). Through conscious effort and choices, individual self‐worth and confidence can increase as individuals learn to live with their illness, benefiting long‐term mental health (Bogart et al. 2012; Brugallé et al. 2021). In this way, RD can be framed as a positive experience, leading to greater acceptance and resilience for future hardships.

### 4.2 Identity Formation and Conflicts

The intersection between mental health and RD has implications for identity formation and conflicts of self, as individuals strive and struggle to define themselves beyond their RD (Nunn 2017; Brugallé et al. 2021). RD inherently shapes the construction of ‘self’, with impacts on family, future goals, peers, and interests (Brugallé et al. 2021). For people living with RD that are lifelong, identity may form around the diagnosis, leading to uncertainty about who they are beyond their condition (Nunn 2017). Whereas for people diagnosed later in life, loss and grief for their previous self and changes in function require that their identity must be reshaped to accommodate the RD (Nunn 2017).

People with RD experience stigma and discrimination and may internalise negative attitudes or judgements (Oksel 2020). Internalised stigma and notions of ‘difference’ or ‘abnormality’ can lead to experiences of ‘othering’—where individuals are marginalised and labelled as intrinsically different from the group (Brugallé et al. 2021; Buckle et al. 2024). Although othering may sometimes be associated with positive experiences such as feeling ‘special’ (Buckle et al. 2024), it mostly elicits negative emotions of hurt, rejection, exclusion, and grief over missed opportunities, compromising mental health (Brugallé et al. 2021). Many adults with RD feel unable to fully define themselves socially without being labelled by their condition (Brugallé et al. 2021). They manage this injustice by forming identities beyond, despite, or alongside their RD and practising acceptance (Hanson et al. 2018). By refusing to be defined by only their RD diagnosis, individuals recalibrate their perception of normality by highlighting their capabilities, rights, and desire to participate as full citizens in their communities (Hanson et al. 2018).

To establish an identity alongside their RD, people must navigate various constraints, including limitations in employment, hobbies, opportunities for self‐expression, and social belonging (Uhlenbusch et al. 2019; Buckle et al. 2024). These factors collectively influence the formation and expression of identity (Uhlenbusch et al. 2019). Adapting to the psychosocial impacts of an RD and its impact on self can cause ‘identity crises’ which can further harm mental health (Munro et al. 2022). Other indirect impacts of RD, such as structural stigmatisation within workplaces, limit opportunities, career progression, and aspirations, contributing to a cycle of hopelessness (Munro et al. 2022). For others, identity formation through RD can be positive, as they take pride in their perseverance and unique perspectives, validating their self‐value (Bogart et al. 2012).

### 4.3 Connection and Disconnection

Social connection impacts mental health, as RD affect how individuals navigate relationships, interactions, and the broader community. While positive experiences of social support can protect mental health, social interactions can often be negative experiences for people with RD and consequently increase social avoidance and disconnection (Bogart et al. 2012). RD impacts social connections through practical restrictions like mobility difficulties, as well as psychological restrictions from stigma, insensitivity and a lack of awareness (Nunn 2017; Uhlenbusch et al. 2019). Many experience discomfort in social contexts related to experiences of social exclusion, intrusive questions, teasing, avoidance, or staring (Henderson et al. 2009; Bogart et al. 2012; Munro et al. 2022; Witt et al. 2023). More traumatic and overt situations of disconnection like mobbing and refusal of access to facilities can also be experienced (Uhlenbusch et al. 2019). Anxiety related to negative social experiences can increase avoidance behaviours and result in further risk of social isolation (Brugallé et al. 2021; Bogart et al. 2012; Munro et al. 2022). However, some individuals are motivated to form new relationships by proactively engaging with others in social situations and seeking to show the ‘person behind the RD’ (Bogart et al. 2012, 1216). Some adopt compensatory strategies such as altering communication and ways of expression to navigate around the practical restrictions of their RD (Bogart et al. 2012).

While many people with RD have meaningful, close relationships and robust support networks, fear of rejection, guilt, or a sense of being a burden can still have a negative impact (Brugallé et al. 2021). Choosing when or whether to explain their RD in new relationships can cause stress and activate feelings of shame and internalised stigma (Bogart et al. 2012). Within existing relationships, individuals may experience frustration and distress related to intrusive questions about diagnoses and symptoms, invalidation and minimisation of its impact, and unsolicited and/or unhelpful advice, (Munro et al. 2022). For people with less visibly ‘obvious’ RD and respective symptoms, for example, chronic fatigue or pain, people may experience cynicism and disbelief about the reality of their disease (Uhlenbusch et al. 2019). This leads to ableist assumptions and judgements of individuals' experience, such as accusations of hypochondriasis, self‐inflicting symptoms or fraudulent attention‐seeking (Munro et al. 2022).

Friends and families are important social supports that are essential for individuals with RD and their mental health and well‐being (Bogart et al. 2012; Henderson et al. 2009). These supportive social relationships provide a safe and comfortable space that enables the expression of identity beyond RD (Bogart et al. 2012). They may provide direct care in RD management and often encourage affected individuals to seek professional support (Henderson et al. 2009; Takeuchi et al. 2016). Supportive friends who are understanding and accepting help establish a sense of ‘normality’ and support feelings of self‐worth (Hanson et al. 2018). Many people find helpful and therapeutic connections in organisations and support groups for others with the same or similar conditions (Munro et al. 2022). Opportunities to connect with others who understand the experience normalises the experience of RD and can be deeply meaningful and positively impact mental health.

### 4.4 Access, Advocacy, and a Lack of Integration

People with RD commonly experience challenges with diagnosis and disease management, which itself can impact mental health. Common challenges include having to manage attendance at multiple health services, delayed diagnosis, misdiagnoses, extensive waiting time, and a lack of clinician knowledge, as well as poor quality or negative experiences of services (Munro et al. 2022; Zhang 2023). Furthermore, there is inadequate integration of services to adequately address both physical and mental health challenges (Takeuchi et al. 2016).

Barriers to accessing health care such as proximity of specialist services, a lack of flexibility in scheduling appointments, struggles in transitioning from paediatric to adult services, and medical expenses hinder individuals from receiving effective care for disease management and addressing emerging or existing mental health conditions (Takeuchi et al. 2016; Nunn 2017; Uhlenbusch et al. 2019; Munro et al. 2022; Witt et al. 2023). Barriers to accessing services can also be specific to the RD—for example, mobility issues or chronic pain can impede an individual's ability to travel and access care (Munro et al. 2022). The barriers and stress associated with navigating multiple healthcare services can also lead to disengagement from treatment, further impacting mental health, well‐being, and quality of life (Hanson et al. 2018; Munro et al. 2022). With RD management and physical care services already challenging to access, engaging with mental health services often becomes a lower priority.

Due to the diverse impacts of RD, multidisciplinary treatments, psychosocial support, and care coordination are essential for effective care (Witt et al. 2023; Zhang 2023). However, there is an apparent lack of service coordination as people with RD commonly adopt the role and responsibilities of care coordination themselves (Nunn 2017; Witt et al. 2023; Zhang 2023). Across studies, many clinicians fail to assess mental health or recommend appropriate support, leading individuals to self‐manage mental health conditions (Witt et al. 2023; Zhang 2023). This requires them to become adept in self‐advocacy, education, and service navigation (Henderson et al. 2009; Munro et al. 2022). When psychosocial or mental health support is accessed, it can be valuable and helpful, but it is often rarely tailored to their unique experience of living with an RD (Witt et al. 2023). Accessing mental health support may be further complicated by the risk of intersectional social stigma and experiences of structural stigma in health services associated with mental illness (Witt et al. 2023), especially as many people with rare diseases have experienced being dismissed as “hypochondriacs” or misdiagnosed with psychosomatic disorders during their diagnostic phase (Nunn 2017). Concerns about experiencing further discrimination and stigma result in individuals also choosing not to access mental health support at all.

Positive interactions between patients and general health service providers can minimise mental distress through trusting relationships and communication. Trustworthiness is enhanced through health professionals showing concern, empathy, and communicating openly and honestly (Witt et al. 2023), leading to individuals feeling more respected, empowered, and understood (Hanson et al. 2018; Munro et al. 2022). However, many patients interact with healthcare professionals who lack knowledge and experience in treating RD, which negatively impacts their experience of care (Uhlenbusch et al. 2019; Witt et al. 2023). Studies highlight that unempathetic, dismissive clinicians and a lack of adequate information during the diagnostic odyssey resulted in growing mistrust in healthcare services (Uhlenbusch et al. 2019; Witt et al. 2023). Individuals with RD also experience other challenges accessing effective care, such as age‐inappropriate resources (Hanson et al. 2018) or assumptions about the impacts of RD on mental health (Witt et al. 2023). Younger individuals may be excluded from healthcare conversations and decisions (Henderson et al. 2009). As a result of these challenges, they often receive compartmentalised and fragmented care, further reducing the quality of care provided and increasing their reluctance to seek professional support.

## 5 Discussion

This narrative review highlights the complex interaction of mental health and RD for people living with a condition, and the common themes of dual experiences. Hopelessness about the future strongly impacts individual's mental health, yet hope and protective experiences of resilience are also apparent. Identity conflicts in the context of RD can be mentally taxing, yet people with RD navigate identity despite, because of, and alongside their RD. Social connections from informal and formal supports are protective for mental health, contrasting experiences of disconnection and isolation from the broader community. Access and coordination of multidisciplinary services and positive patient‐clinician relationships encourage patient advocacy and collaborative decision‐making. Thus, while mental distress is common for the RD community, many also overcome challenges and retain hope. The findings have implications for understanding the intersection between mental health and RD, as well as the need for integrated service delivery.

A fundamental aspect of having an RD is living with a long‐term, often misunderstood, and not well‐known disorder characterised by uncertainty, which is linked to negative mental health outcomes (Henderson et al. 2009; Massazza et al. 2023). Uncertainty is known to be associated with the diagnostic odyssey and disease management. However, current findings highlight its pervasive influence over career, family planning, identity formation, and social connection—leading to hopelessness and distress. These findings are congruent with existing literature, which highlights uncertainty about disease evolution, future planning and treatment (von der Lippe et al. 2017; Llubes‐Arrià et al. 2022; Bauskis et al. 2022; Kenny and Stone 2022). The review findings position uncertainty as a challenge, but also an opportunity for identity formation, resilience and empowerment. This resonates with Mishel's theory of ‘uncertainty in illness’ (1990), which builds on the transactional model of stress and coping of Lazarus and Folkman (Lazarus and Folkman, as cited in Mishel 1990). Through the continual uncertainty associated with sustained health conditions, destabilisation causes people to reformulate their view of life and self, promoting resilience and perseverance (Rogers and Walker 2016). The review identifies instances of individuals with RD priding themselves on their diversity and tolerance to adversity, reflecting their ability to validate uncertainty and strive beyond it. In Mishel's model (Mishel 1990), resilience is also relational, with social and healthcare resources facilitating the capacity to use uncertainty as a positive force. In our findings, the significance of social support and positive patient‐provider relationships were seen as protective factors for mental health, although some may lack the understanding of unique RD uncertainties. The complex influence of uncertainty with resilience, hope and hopelessness, and the implications for identity, lies at the intersection of mental health and RD.

Underpinning all the themes identified in this review from the intersection of mental health and RD, were experiences of stigma. Health‐related stigma, characterised by negative social processes based on identifying health concerns, was the most prevalent (Scambler 2009). This aligns with existing literature where incidents of marginalisation and discrimination for people with RD are well‐documented (Llubes‐Arrià et al. 2022; von der Lippe et al. 2017; Kenny and Stone 2022; Wiegand‐Grefe et al. 2022). The current review highlights internalised shame, othering and experiences of social exclusion either directly or indirectly caused by RD, which all contribute to mental distress. The review also highlights the compounding intersectional stigma of two experiences—RD and mental health. Mental health stigma has been well‐researched (Wallace 2012; Corrigan et al. 2014; Henderson et al. 2014), however, intersectional stigma for people with RD and mental health challenges has not (Turan et al. 2019). This review found that stigmatising experiences of misdiagnoses and blame during the diagnostic phase led to a reluctance to actively engage in any help‐seeking for fear of perpetuating judgement, especially seeking for mental health support. Individuals with RD experience of intersecting identities were impacted by a lack of awareness of RD, even within health services, and negative attitudes towards mental disorders, leading to self‐stigma, shame and decreased resilience (Crowe et al. 2016; Turan et al. 2019). In response to stigma, individuals downplay or hide to facilitate adjustment, whilst some may overcome stigma by actively embracing their identity (Scambler 2009). The review highlighted this, contrasting people who adopted passive, restrictive, or socially isolating behaviours, and those who engaged in self‐advocacy, educating others, and raising awareness of RD. While experiences of stigma at the intersection of mental health and RD align with findings from other health fields, the unique, intersecting stigma of these two experiences remains poorly explored with bi‐directional impacts upon both.

The themes of hope, resilience, identity, overcoming stigma, and finding meaning and self in the context of sustained experiences of RD have parallels with the concept of personal recovery in mental health. Personal recovery emphasises a journey of learning to live meaningfully alongside the constraints of illnesses, conceptualised through the CHIME framework (Connectedness, Hope, Identity, Meaning, and Empowerment) (Bird et al. 2014). Features of CHIME were apparent throughout the review in the similarities experienced by individuals dealing with and overcoming the challenges of mental illness and RD. Protective and supportive factors were highlighted through social support, RD patient organisations, and positive healthcare experiences, which foster hope and the development of effective coping strategies. As with personal recovery (Slade 2009), these factors also assist with overcoming stigma, allowing identity to be redefined in the context of RD, and establishing a meaningful life despite limitations. However, while existing RD literature commonly mentions resilience and empowerment, the dominant deficit‐based narrative continues to prevail. The current review highlights the importance of a strength‐based approach, in which the values of autonomy and empowerment underpin the treatment approach.

The findings of this review indicate an urgent need for accessible and integrated care across physical and mental health services for people with RD. The reciprocal relationship between physical and mental health is well established in literature across fields such as oncology, palliative care, and chronic care (Turner and Kelly 2000; Carta et al. 2017; Pitman et al. 2018). Greater severity of physical illness, regardless of the underlying cause, is commonly associated with the frequency and severity of depressive symptoms and can be a determinant of resilience and will to live (Spiegel and Giese‐Davis 2003). Similarly, people experiencing symptoms of mental illness are also at an increased risk of physical illness and premature mortality (Robson and Gray 2007; Ohrnberger et al. 2017). Despite this, across the findings, psychosocial support was positioned as sidelined, with priorities placed on physical symptom control. In other health fields, such as oncology, where supports like psychotherapies and cognitive/behavioural restructuring have long been integrated into standard medical treatment (Spiegel and Giese‐Davis 2003), there is a lack of understanding and multidisciplinary application within the services assisting people with RDs. Poor integration is further complicated by accessibility barriers, where the review outlined both systemic service‐based deficiencies alongside inherent physical and emotional challenges of RDs impeding individuals' ability to access care. Aspects of accessibility, such as poor affordability and lack of available services, to the inhibited ability for people to perceive the need for care and to reach and engage in supports, were all identified in the study. Even when people were able to recognise the need for support, the compounded challenges of poor accessibility and lack of integrated care forced them into a role of self‐advocacy and informal support seeking. This then perpetuated an ongoing cycle of unwillingness to seek future formal support (Witt et al. 2023; Zhang 2023). These challenges parallel disparities in other specialised care domains for RDs, like palliative care for rare cancers (Kostadinov et al. 2025). Discrepancies in public health expenditure were a significant influence on accessibility and perceived quality of care, with disparities in funding mechanisms reflecting similar disparities in equitable care delivery (Kostadinov et al. 2025). Evidently, systemic deficiencies impact the availability and accessibility of quality and tailored care for the vulnerable population of people with RDs, who face unique and compounding vulnerabilities like diagnostic complexities and inherent distress. This indicates a gap in awareness and recognition of the need to improve mental health care delivery for people with RD on both micro‐ and macro‐levels, impeding the delivery of integrated, recovery‐oriented care.

### 5.1 Limitations

Although constructed through robust methods, this review presents one possible narrative of the diverse experiences under the umbrella of RD and mental health. The lack of research across their intersection means included literature will not reflect all individuals with RDs and their experiences. Whilst the inclusion criteria outlined literature where authors are people with RDs, it is also acknowledged that people with lived experience may not simultaneously be academics and may be published as grey literature outside of academic databases. As the study exclusively scoured articles on research databases, lived experience literature may hence be indirectly excluded.

The bulk of literature on RD also centres around paediatric RD with a focus on caregiver and parental experiences, leading to a conflation of their perspective on psychological burden. While this review focused generally on the affected people with RD, caregiver experiences may have influenced the findings when discussing paediatric patients.

Due to the wide variety and number of RD, the process of systematically searching the literature was complex, as many studies used specific RD names rather than the collective term. With over 6000 RD, it was not feasible to explore all conditions. This means that the review may be biased towards more “well‐known” RDs and the experiences of the conditions explored in the included research papers. It can, however, be assumed that the intersection of mental health and other RD is likely plagued by similar experiences.

The review included studies from across the globe over an extensive time period, which may impact finding specificity and transferability, as experiences have occurred in differing contexts and may not align with current practices. As different healthcare systems incorporate varied scopes of clinical practice, systemic policies, definitions of RDs, service structures, and funding, these contextual factors inherently shape individual experiences and transferability of the findings.

## 6 Conclusion

This narrative review explored the intersection between mental health and RD through lived experiences, revealing how mental distress and resilience are deeply integrated into the experience of living with RD. People with RD navigate the uncertain, anxiety‐inducing, and stressful landscape of disease management while maintaining resilience and hope. The need to reshape life and identity in the context of RD triggers moments of crises and grief, as individuals learn to persevere despite limitations. With incidences of stigmatisation and discrimination, social isolation and a disconnect from the community are not uncommon. However, positive relationships benefit mental well‐being. Having access to appropriate and sensitive health services is vital in supporting individuals through the physical and mental challenges associated with their disease journey. The complex journey of navigating mental well‐being alongside RD is a process of personal recovery for individuals, with challenges of uncertainty and stigma. A need for an in‐depth understanding of local service contexts and enablers of supportive care is evident.

## Author Contributions

**Eileen Wu:** conceptualisation, data curation, analysis, investigation, methodology, project administration, writing – original draft. **Sophie Isobel:** conceptualisation, methodology, supervision, writing – review and editing. **Paul Beckett:** conceptualisation, methodology, supervision, writing – review and editing.

## Funding

The authors have nothing to report.

## Disclosure

All authors meet the authorship criteria according to the latest guidelines of the International Committee of Medical Journal Editors.

## Conflicts of Interest

The authors declare no conflicts of interest.

## Data Availability

Data sharing not applicable to this article as no datasets were generated or analysed during the current study.

## Appendix A

### Search strategies utilised for literature search

**Table:** TABLE A1. Search strategy ‐ CINAHL.

	Major Heading	Rare Diseases”
	Search terms	OR ((rare* or orphan*) N3 (disease* or disorder*))
AND	Major Heading	"Mental Health" OR "Mental Health Organizations+" OR "Mental Health Personnel+" OR "Mental Health Counseling" OR "Mental Health Teletherapy" OR "Mental Health Services+" OR "Mental Health (Omaha)" OR "Community Mental Health Nurses" OR "School Mental Health Services" OR "Community Mental Health Nursing” OR "Mental Health Care (Saba CCC)+" OR "Hospitals, Psychiatric" OR "Psychiatric Nursing+"
	Search terms	OR “mental health” or psychosocial
AND	Search terms	experience* OR intersection* OR perspective* OR care* OR access* OR use* OR usag* OR support* OR utilis* OR utiliz* OR "lived experience" OR "living with"

**Table:** TABLE A2. Search strategy – SCOPUS.

	Article title, Abstract, Keywords	(rare OR orphan) w/3 (disorder* or disease*)
AND	Article title, Abstract, Keywords	“mental health” OR psychosocial
AND	Article title, Abstract, Keywords	experience* OR intersection* OR perspective* OR care* OR access* OR use* OR usag* OR support* OR utilis* OR utiliz* OR “lived experience” OR “living with”OR service*
Limited to English, Excluded Review/Book chapter/Conference paper.

**Table:** TABLE A3. Search strategy – PUBMED.

	MeSH Major Topic	Rare Diseases
	[Title/Abstract]	OR “rare* disease*” OR “rare* disorder*” OR “orphan* disease*” OR “orphan* disorder*”
AND	MeSH Major Topic	Mental Health
	[Title/Abstract]	OR “mental health” OR psychosocial
AND	[Title/Abstract]	experience* OR intersection* OR perspective* OR care* OR access* OR use* OR usag* OR support* OR utilis* OR utiliz* OR “lived experience” OR “living with” OR service*

**Table:** TABLE A4. Search strategy – Medline.

	Subject Heading	Rare Diseases
	Keyword	OR ((rare* or orphan*) adj3 (disorder* or disease*))
AND	Subject Heading	Mental health/OR Mental disorders/
	Keyword	OR “mental health” OR psychosocial
AND	Keyword	experience* OR intersection* OR perspective* OR care* OR access* OR use* OR usag* OR support* OR utilis* OR utiliz* OR “lived experience” OR “living with” OR service*
Limit to English language.

**Table:** TABLE A5. Search strategy – Embase.

	Subject Heading	Rare disease
	Keyword	OR ((rare* or orphan*) adj3 (disease* or disorder*))
AND	Subject Heading	Mental health/OR Community mental health/OR Mental health care/OR Mental health care access/OR Mental health care personnel/OR Mental health center/OR Mental health organisation/OR Mental health service/OR Mental hospital
	Keyword	OR “mental health” OR psychosocial
AND	Keyword	experience* OR intersection* OR perspective* OR care* OR access* OR use* OR usag* OR support* OR utilis* OR utiliz* OR “lived experience” OR “living with” OR service*
Limit to English Language, full text.

**Table:** TABLE A6. Search strategy – PsycInfo*.*

	Keyword	(rare* or orphan*) adj3 (disease* or disorder*)
AND	Subject Heading	Mental health/OR Youth mental health/OR Community mental health services/OR Community psychiatry/OR Mental disorders/OR Mental health services/OR Psychosocial outcomes/OR Mental health personnel/OR Mental health programs
	Keyword	OR “mental health” or psychosocial
AND	Keyword	experience* OR intersection* OR perspective* OR care* OR access* OR use* OR usag* OR support* OR utilis* OR utiliz* OR “lived experience” OR “living with”
Limit to peer reviewed journal, English language.		
